# Association among Household Wealth, Maternal Employment, and Undernutrition in Children under Three Years of Age in Pakistan

**DOI:** 10.3390/children11070872

**Published:** 2024-07-18

**Authors:** Muhammad Shahid, Yuantao Xie, Shamshad Bashir, Nazia Noureen, Jiayi Song, Najma Iqbal Malik, Kun Tang

**Affiliations:** 1 School of Insurance and Economics, University of International Business and Economics, Beijing 100029, China; de202159006@uibe.edu.cn (M.S.); xieyuantao@uibe.edu.cn (Y.X.); 2 Department of Psychology, Lahore Garrison University, Lahore 54000, Pakistan; shamshadbashir@lgu.edu.pk; 3 Department of Psychology, Foundation University Rawalpindi Campus, Rawalpindi 44000, Pakistan; nazia.ch88@gmail.com; 4 Tsinghua Shenzhen International School, Tsinghua University, Beijing 100084, China; 5 Department of Psychology, University of Sargodha, Sargodha 40100, Pakistan; najma.iqbal@uos.edu.pk; 6 Vanke School of Public Health, Tsinghua University, Beijing 100084, China

**Keywords:** undernutrition, stunting, underweight, wasting, women employment, household wealth, Pakistan

## Abstract

Background: There is an abundance of studies explaining the separate impact of female employment and household wealth status in reducing malnutrition. However, our study has unraveled the combined impact of maternal employment and household wealth on undernutrition among children under three in Pakistan. Methods: Using a sample of 1093 children under three years of age from the Pakistan Demographic and Health Survey 2017–2018, a binary logistic model was employed to gauge factors influencing the children’s undernutrition. Results: Our results indicated that children up to a certain age (three years old) with residence in certain regions (Pakistan) and recent episodes of diarrhea had an increased risk of undernutrition. Conversely, secondary and higher maternal education, access to improved water sources, and sanitation facilities lowered the chances of undernutrition in children under three in Pakistan. The interaction between maternal employment and household wealth showed that maternal employment significantly lowered the risk of stunting, being underweight, and wasting among the average, rich, and richest households; however, it did not contribute to child nutrition among the poorer and poor households. Notably, regardless of whether the mother was employed, the wealth status of being rich and richest reduced the risk of stunting, being underweight, and wasting. Conclusions: In overcoming undernutrition, maternal employment significantly contributed to middle-income households. However, in the richer and richest households, the wealth status played a more crucial role compared to the maternal employment. This indicates that while employment plays a supportive role in household resources, the wealth status is overall more influential in reducing undernutrition.

## 1. Introduction

Undernutrition among young children poses a critical public health concern, particularly in less developed nations [1,2,3]. In regions like Asia and Africa, children commonly suffer from stunting, wasting, and being underweight [4,5]. Countries such as Pakistan, India, and Bangladesh grapple with undernutrition rates surpassing the established thresholds, with stunting affecting over 30%, wasting around 15%, and being underweight impacting approximately 10% of the child population [5,6,7]. The repercussions are severe, as malnourished children face a significantly elevated risk of mortality, ranging from 4 to 12 times higher compared to well-nourished children [8].

The socioeconomic status of households plays a central role in determining the nutritional outcomes, encompassing various factors such as sanitation, access to safe drinking water, women’s education, household wealth, and employment status [9]. A lower socioeconomic status has been closely linked with negative health and nutrition outcomes, which can be attributed to factors such as limited educational attainment, unemployment, and reduced economic purchasing power [10,11].

Numerous studies carried out across various regions like China, Mexico, and Sub-Saharan Africa have consistently highlighted the heightened susceptibility of children from economically disadvantaged backgrounds to stunting [12,13,14]. Pakistan is among the nations facing ongoing challenges with chronic malnutrition, especially among children below the age of five [15]. Research conducted within Pakistan has indicated substantial decreases in the prevalence of stunting and being underweight when households transition from the lower to middle and higher socioeconomic strata [16,17].

The influence of undernutrition on children’s development varies based on their socioeconomic context and the particular type of malnutrition that they encounter [18]. As an example, children who experience stunting often come from households that earn significantly lower wages, estimated at less than 20% of their non-stunted counterparts, and face a higher risk of living in poverty, surpassing 30% [19]. Malnutrition imposes a significant economic burden on underdeveloped nations, leading to annual reductions in gross domestic product (GDP) [20]. Stunting and being underweight, recognized as the primary facets of malnutrition, contribute to an estimated 11% of GDP losses in Asian and African regions [21]. Investing in nutrition programs has demonstrated significant economic benefits, with an estimated return of USD 30 for every USD 1 invested in the efforts aimed at reducing stunting in underdeveloped nations [22].

In many developing countries, women commonly juggle dual responsibilities, both caring for their children and contributing to the household income. Traditionally, men have predominantly been the primary earners and less involved in childcare, particularly for children under the age of five. Mothers typically take on the primary caregiving role, but when they work, they may struggle to find enough time for childcare. Employed mothers often rely on extended family members to look after their children in their absence, potentially resulting in less-than-optimal care in terms of both quality and quantity. Consequently, working mothers may face a trade-off in the form of reduced time for childcare. On the other hand, the increased household income resulting from women’s employment may unintentionally lead to insufficient childcare due to time constraints [23]. Building upon these foundations, this study aims to determine whether working mothers experience a trade-off in terms of diminished childcare. Specifically, the research delves into examining how female labor force participation, alongside their socioeconomic status, collectively influences the nutritional well-being of children under the age of three in Pakistan. By doing so, this research endeavors to address a notable gap in the current understanding within the unique context of Pakistan.

## 2. Materials and Methods

### 2.1. Theoretical Framework

A significant portion of the existing literature on child undernutrition draws upon the utility maximizing model, which incorporates the household’s production function [24,25]. Beaker had used the household production function for health, so the objective of this study is to replicate this model for children’s health (i.e., for nutritional outcomes). 

The inputs in Beaker’s health production function includes the following: consumption, which means good nutrition/food intake (the main source of energy), medical care, environmental factors such as water and sanitation, and household and individual factors. Beaker assumed that a household would have the following utility function:

(1) U=U (H,X,C,LP,LO,He, E, Z)

where utility *U* depends on the current health status of the household members “*H*”; consumption of food and drink “*X*”, other purchased goods “*C*” (excluding purchased health care); and physically active leisure “*LP*” and other leisure time “*LO*”. The variable “*He*” represents early health status, e.g., genetic potential for good/bad health or sometimes summarized by health status at birth such as birth weight. “*E*” represents the environmental factors such as water and sanitation. “*Z*” denotes fixed observables factors or household or individual factors, such as education, employment, wealth, gender, age, and race–ethnicity of adults. The household would want to maximize that utility subject to a budget constraint such as the following:

(2) PH+PX+PC+PLP+PLO+PHe+PE+PZ ≤ W

where “*P*” are the prices of above factors and “*W*” total income of the household. The marginal increase in any one of the above indicators directly increases household utility *U* > 0, and better (current) adult health status increases the household utility. There is a further replication of this household production function in this study where the children’s nutritional status is a dependent variable. Assuming the household’s production function for the nutritional status of children under three is as follows:

(3) Ni=N(X,He, E, Z) 

where “*Ni*” is used as the standard measurement of anthropometry for a child’s nutritional status which is comprised of underweight (WAZ), wasting (WHZ) and stunting (HAZ); “*X*” is the consumption of food and drinks; “*He*” represents early health status, e.g., genetic potential for good/bad health (diseases such as diarrhea or birth weight, etc.); “*E*” denotes environmental factors such as water and sanitation; “*Z*” denotes fixed observables factors or household or individual factors, such as education, maternal employment, household wealth, gender, age, area of residence, and region, etc.; and the parameter σ summarizes unobservable factors which affect the efficiency of the current production of children’s nutritional status. In conclusion, it shows that provision in the above socio-economic factors increases the nutritional status of children.

### 2.2. Data and Description

**Figure 1:**
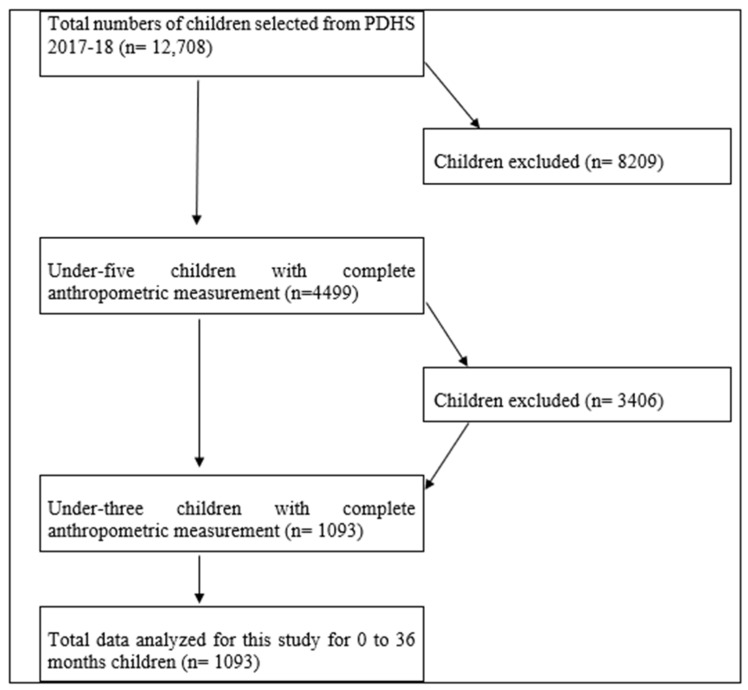
Flow Chart of Study Sample. Source: Authors.

This study utilized data collected from the Demographic and Health Survey (PDHS) performed in 2017–2018. The data of 12,708 children in total were given in the PDHS survey, but the children with complete anthropometric measurements were only 4499. As the study target group was children under three, the study excluded 3406 children from the analysis. Finally, the survey considered a sample of 1093 children under the age of three for the final analysis, as explained in Figure 1 (flow chart of study sample). The dataset contains a comprehensive range of information on nutrition and demographic characteristics, as well as data on medical care, nutrition status, women’s empowerment, occurrences of domestic violence, and various other relevant factors. Statistical analyses were conducted using anthropometric measurements obtained from eligible children under three years of age. Additionally, the study incorporated data on household characteristics, specific variables related to children and mothers, factors related to child health, and various environmental and socioeconomic attributes.

### 2.3. Response Variable of the Study

The research utilized data from the PDHS 2017–2018 to assess child undernutrition by using three anthropometric measurements, as these indices (HAZ, WAZ, WHZ) were based on the growth standards established by the World Health Organization (WHO) in 2009 [26]. The study has defined the following: (i) stunting as “height-for-age z-scores (HAZ) falling below −2 standard deviations (S.D) from the median value based on WHO criteria”; (ii) being underweight as “weight-for-age z score (WAZ) falling below −2 S.D. of the median value according to WHO standards”; and (iii) wasting delineated by “weight-for-height z-scores falling below −2 S.D from the median value as per WHO guidelines”. PDHS 2017–2018 used information on height, weight, and age for eligible children to construct these three indices. However, stunting, being underweight, and wasting were transformed into binary variables, with stunting being assigned 0 and 1, where 1 implied “stunting” and 0 meant “not stunting”. Being underweight and wasting were also defined in a same way, where 0 was coded for “not underweight or not wasted”, and 1 meant “underweight or wasted”.

### 2.4. Data Analysis

Throughout this study, a hypothesis was formulated suggesting that several socio-economic indicators have an impact on undernutrition among children under three. To explore the relationship between the proximate factors (particularly women employment status and household wealth status, and dependent variable (children’s undernutrition), a binary logistic regression analysis was chosen as the preferred analytical method. This study used the three indices of undernutrition assessment, i.e., stunting, underweight, and wasting as dependent variables, to comprehensively evaluate the nutritional well-being of children. The binary logistic regression is explained as follows:

$$\begin{array}{ll} {Undernutrition}_{i} & =Y_{i}\\ & =(1\;if\;the\;child\;is\;stunted/wasted/\;underweight,\;0\;if\;the\;child\;is\;not\;stunted/not\;wasted\\ & /not\;underweight) \end{array}$$

The binary logistic model is explained as follows: The variables “Undernutrition” and *Y_i_* are inherently binary, with a value of “1” indicating the presence of any type of undernutrition (stunting, wasting, underweight) among children, while the value “0” implies the absence of undernutrition. In this binary response framework utilized in this context, a clear distinction between success and failure is established, where success represents child undernutrition and failure represents the absence of undernutrition. However, within the scope of this research, the variable *Y* represents the nutritional statuses of the children and is examined in relation to several regressors denoted as *X*.

This study proceeds by outlining the model specifications as well as the reduced form of the binary logistic regression. This statistical technique aims to determine odds ratios associated with stunting, underweight, wasting, representing children’s undernutrition, while accounting for a variety of explanatory variables. The formulation of this regression model is articulated as follows:

$$Stunting/underweight/wasting=(\alpha_{0}+\alpha_{1}X_{1i}+\alpha_{2}X_{2i}+...+\alpha_{n}X_{kn})$$

The equation incorporates multiple variables and coefficients aimed at exploring the relationship between the response variables (stunting, wasting, underweight) and regressors denoted as *X_i_*. The *α* coefficients quantify the strength of association among dependent variables such as stunting, wasting, and being underweight, and other regressors. Additionally, the inclusion of the error term ε is crucial to accommodate any unaccounted-for variability within the model.

## 3. Results

### 3.1. Descriptive Statistics

This paper analyzed the trends of undernutrition among children under three years of age in descriptive analysis. 

**Figure 2:**
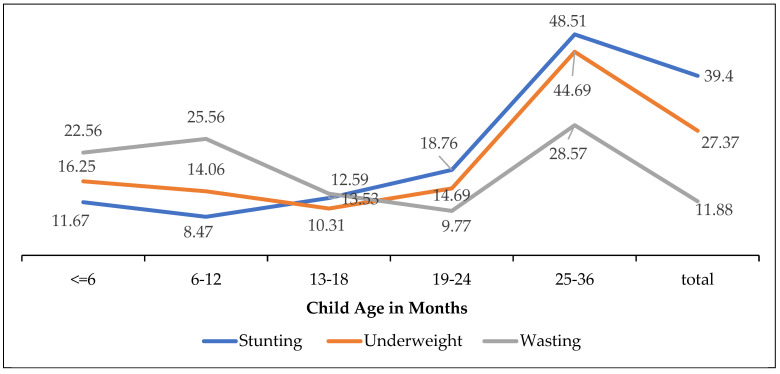
Undernutrition among children under three years age group. Source: Authors.

In Figure 2, which shows the undernutrition trend, stunting prevalence was lower in the age group of 6–12 months; however, it showed a steady increase until the age of 19–24 months, and after that, the age group stunting significantly increased until the age of 25–36 months. Similarly, the prevalence of being underweight decreased until the age of 13–18 months but then significantly increased until the age group 25–36 months. Wasting prevalence increased until the age range of 6–12 months, and then it decreased in the age group of 19–24 months. However, it proceeded to increase again until the age of 25–36 months. 

**Figure 3:**
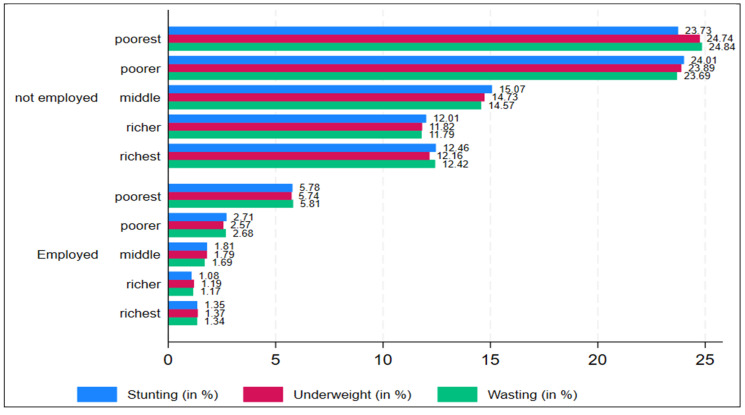
Undernutrition prevalence among children under three years age group across women employment by household wealth Index. Source: Authors.

Figure 3 visually depicts the trends in undernutrition prevalence over wealth status by women in employment. The prevalence of stunting, being underweight, and wasting are significantly high among the poorest and poorer households where women are not employed, while after that, the rates of undernutrition prevalence decreased in the middle income, richer, and richest households where the women were not engaged in any employment. Similarly, the prevalence of undernutrition was high among children in the poorest, poorer, and middle wealth households where the women were employed. Moreover, the prevalence of stunting, being underweight, and wasting decreased in the richer and richest households where the women were engaged in any employment. Overall, the prevalence of stunting, being underweight, and wasting was significantly high among the households where women were not employed.

The descriptive results of the chi-squared test are presented in Table 1. The prevalence of undernutrition was highest in males (51.72% stunting; 51.88% underweight; and 54.89% wasting) among the children under three in Pakistan. The undernutrition prevalence was high in the age group of 25–36 months (48.51% stunting; 44.69% underweight; and 28.57% wasting) among the children under three. The stunting prevalence was high among children in birth order one (39.82%) and birth order two (27.92%). The underweight prevalence was also high in birth order one (34%) and birth order two (30.31%). Similarly, the wasting rates were also high in birth order one (33%) and birth order two (36%). The undernutrition prevalence was high among the children under three (86.14% stunting; 83.23% underweight; and 89.39% wasting) for undernourished mothers with a BMI of <18.5 kg/m^2^. The prevalence of undernutrition was high among the children under three with illiterate mothers (75% stunting; 79.38% underweight; and 73.68% wasting). Prevalence of undernutrition was high among the children who belonged to rural areas (64.30% stunting; 66.565% underweight; and 63.16% wasting). The prevalence of undernutrition was high in two less developed regions of Pakistan, which were Sindh (24.94% stunting; 30.31% underweight; and 22.56% wasting) and Balochistan (22.43% stunting; 29.69% underweight; and 33% wasting). The prevalence of undernutrition was high among the households with unimproved water facilities (71.85% stunting; 70.62% underweight; and 72.93% wasting) and households with unimproved sanitation facilities (60.64% stunting; 60.94% underweight; and 68.42% wasting).

The undernutrition prevalence was high among children who had experienced episodes of diarrhea (73.23% stunting; 75% underweight; and 75.94% wasting). The prevalence of undernutrition was high among children belonging to the poorest wealth quantile (41.19% stunting; 45.31% underweight; and 33% wasting), and poorer wealth quantile (28.38% stunting; 28.12% underweight; and 34.59% wasting). The undernutrition prevalence was high among the children of mothers who were not employed (85.13% stunting; 83.44% underweight; and 87.22% wasting). These chi-squared findings highlighted a multitude of demographic and socioeconomic variables that play crucial roles in undernutrition prevalence among younger children.

### 3.2. Binary Logistic Regression Estimates

Table 2 provides a comprehensive overview of binary logistic regression output, furnishing valuable insights into the factors associated with child undernutrition. Across the children’s age groups, the adjusted odds were higher among the age group of 19–24 months and 25–36 months for stunting [19–24 months (AOR = 2.56, 95% CI: 1.61–4.07); and 25–36 months (OR = 1.94, 95% CI: 1.80–3.80)], for underweight [19–24 months (AOR = 1.64, 95% CI: 1.98–2.74); and 25–36 months (OR = 1.83, 95% CI: 1.22–2.76)], and for wasting [19–24 months (AOR = 1.50, 95% CI: 1.24–1.47); and 25–36 months (OR = 1.03, 95% CI: 1.33–1.96)]. It is noteworthy to emphasize the presence of a non-linear relationship with age, indicating that stunting, being underweight, and wasting prevalence increases until a specific age threshold, following a gradual decline afterward. The research outcomes underscore the significant roles of maternal secondary and higher education in alleviating the likelihood of child undernutrition. The odds of stunting prevalence were lower among the children under three with mothers who had completed secondary (OR = 0.57, 95% CI: 0.42–0.86) and higher education (OR = 0.32, 95% CI: 0.19–0.55). It is worth noting that substantial regional disparities were discernible, notably in the Sindh province, where children under the age of three faced higher odds of experiencing stunting (AOR = 2.57, 95% CI: 1.71–4.41), being underweight (AOR = 3.55, 95% CI: 2.02–6.27), and wasting (AOR = 1.75, 95% CI: 1.82–3.73). Elevated odds of stunting (OR = 1.73, 95% CI: 1.07–2.81), higher odds of being underweight (OR = 2.04, 95% CI: 1.11–3.74), and higher odds of wasting (OR= 1.89, 95% CI: 1.88–4.05) were observed in the Khyber Pakhtunkhwa province. Similarly, the odds were also higher among children in the case of stunting (OR = 2.98, 95% CI: 1.82–4.91), being underweight (OR= 3.48, 95% CI: 1.94–6.22), and wasting (OR = 3.04, 95% CI: 1.43–6.45) in the Balochistan province. Households with improved water sources were associated with lower odds of children under three experiencing stunting (OR = 0.65, 95% CI: 0.47–0.92), being underweight (OR = 0.53, 95% CI: 0.37–0.76), and wasting (OR = 0.70, 95% CI: 0.08–0.82). Similarly, households with improved sanitation facilities were also associated with lower odds of stunting (OR = 0.55, 95% CI: 0.72–0.99), being underweight (OR = 0.23, 95% CI: 0.76–0.86), and wasting (OR = 0.29, 95% CI: 0.35–0.43). Children experiencing episodes of diarrhea were associated with higher odds of stunting (OR = 1.41, 95% CI: 1.04–1.90), being underweight (OR= 1.15, 95% CI: 1.28–1.62), and wasting (OR = 1.94, 95% CI: 1.49–1.59). The gender of the child, order of childbirth, mother’s BMI, and area of residence were not statistically significant for stunting, being underweight, and wasting. 

Furthermore, the results for the interaction terms of household wealth status and women working status depicted that the odds were lower for stunting (OR = 0.59, 95% CI: 0.22–0.64), being underweight (OR = 0.91, 95% CI: 0.37–0.48), and wasting (OR = 0.73, 95% CI: 0.40–0.75) among the children whose mothers had employment and whose household had an average/middle wealth status. In the middle wealth status household’s interaction category, female employment had a significant impact on child nutritional outcomes. Results further depicted that the odds of undernutrition were lower in the richer [stunting (OR = 0.41, 95% CI: 0.25–0.69), underweight (OR = 0.49, 95% CI: 0.25–0.94), and wasting (OR = 0.86, 95% CI: 0.37–0.45)] and richest households [stunting (OR = 0.35, 95% CI: 0.19–0.62), underweight (OR = 0.25, 95% CI: 0.10–0.58), and wasting (OR = 0.57, 95% CI: 0.20–0.58)] when the women were not engaged in any employment. In these two interaction categories, household wealth status had a more significant impact compared to the women’s employment status. The results further depicted that the chances of undernutrition decreased if the household belonged to the richer [stunting (OR = 0.46, 95% CI: 0.40–0.32), underweight (OR = 0.83, 95% CI: 0.20–0.36), and wasting (OR = 0.61, 95% CI: 0.33–0.57)] and richest wealth status groups [stunting (OR = 0.17, 95% CI: 0.03–0.87), underweight (OR = 0.21, 95% CI: 0.05–0.21), and wasting (OR = 0.67, 95% CI: 0.06–0.86)] when the mother was employed. This shows that maternal employment gives extra support to family resources in the richer and richest households which, in the end, significantly impacts child nutrition.

**Figure 4:**
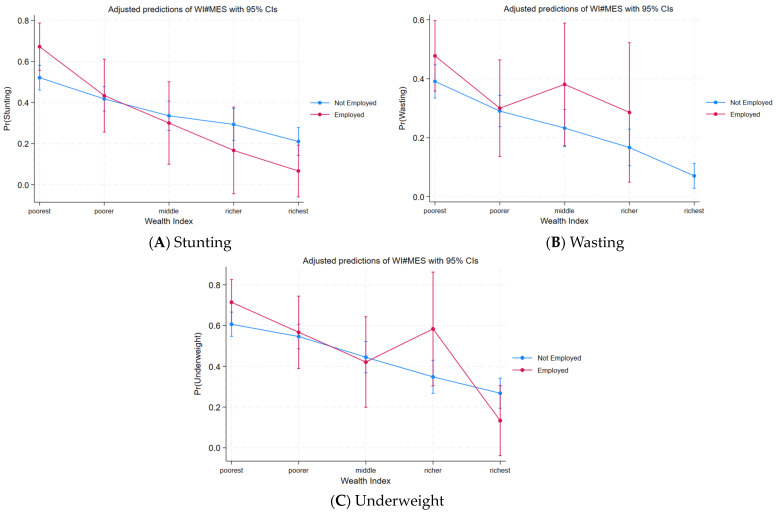
Projected probabilities of undernutrition prevalence by women employment status and household wealth status. Source: Authors.

Figure 4A–C present the projected probabilities of undernutrition in relation to the women’s employment status and household wealth status, based on the binary logistic regression model. They depict that the probability of stunting, wasting, and being underweight continuously decreased with the increase in wealth status from poorer to richest wealth status when women were not employed. It can be seen that if the women are not employed and remain in the household as a housewife, they can give better care and time to their children. Figure 4A–C further depict that when women are employed, the probability of stunting continuously decreases in all wealth statuses, while in the case of being underweight and wasting, it decreases until the middle wealth status, following which it shows an increase in the richer household. The increased projected probability of undernutrition in richer households may have been due to the employed mother facing opportunity costs in the form of a reduction in their children’s care.

### 3.3. Post-Analysis Estimation for Model Performance

The study performed sensitivity and specificity tests for the classification of the performance of the binary logistic regression model. Table 3 shows the results of sensitivity and specificity for stunting, being underweight, and wasting. It shows that using these given set of explanatory variables, the outcome variables such as stunting, being underweight, and wasting are classified by 69.48%, 75.07%, and 88.03%, respectively, which means that the model’s performance is very good.

**Figure 5:**
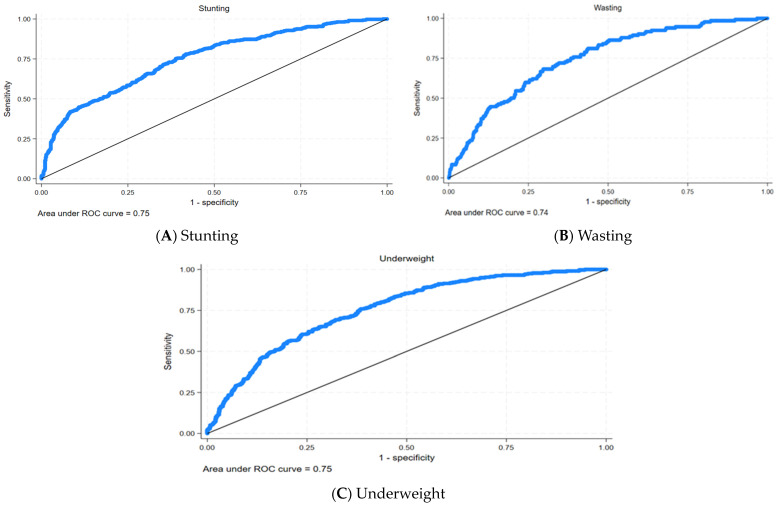
ROC curves for undernutrition. Source: Authors.

Similarly, Figure 5A–C represent the ROC curves for undernutrition in the children. In the ROC curve, the closer the area is to 1, the better the performance of the model. Figure 5 shows that the area under the ROC curve is 0.75 for stunting and being underweight, and 0.74 for wasting, which means the model is a good fit.

## 4. Discussion

The findings indicated that the prevalence rates of stunting, underweight conditions, and wasting were 39.40%, 27.37%, and 11.88%, respectively. Furthermore, the results revealed a positive correlation between the age of the child and the likelihood of stunting, being underweight, and wasting, which aligns with the existing studies [27,28,29,30]. Nonetheless, this relationship demonstrated a nonlinear pattern, with undernutrition prevalence increasing up to a certain age threshold and subsequently diminishing. The inverse correlation observed between the square of the children’s age and the stunting, wasting, and underweight conditions suggests a decline in undernutrition risk beyond a specific age, potentially attributed to reduced exposure to unhygienic conditions during teething and crawling. Thus, the heightened vulnerability of younger children to infections has been identified as a contributing factor to their increased risk of undernutrition.

Regionally, it was found that children residing in Sindh, Balochistan, and KPK exhibited a significantly higher likelihood of experiencing undernutrition (stunting, being underweight, and wasting). These regional disparities in nutritional status have been well-documented in the context of Pakistan, with women in the Sindh, Balochistan, and KPK regions exhibiting higher levels of undernutrition prevalence during adulthood or earlier life stages. The prevailing socioeconomic deprivation in these regions contributes to the elevated prevalence of undernutrition-related conditions [31]. Another study illustrated that undernutrition rates in the Sindh and Balochistan regions surpassed those in other regions of Pakistan, attributed to the underdeveloped nature of these areas [32]. Additionally, this study emphasized the importance of recent episodes of diarrhea, which were found to significantly increase the risk for children to be affected by stunting, being underweight, and wasting. This finding is consistent with the previous research, highlighting that children who have recently experienced diarrhea are more prone to undernutrition [33,34,35].

Maternal education emerged as a crucial factor in reducing the risk of child stunting, wasting, and being underweight, particularly at the secondary and postsecondary education levels. While primary and secondary education alone did not seem to have a significant impact on nutritional status, higher levels of maternal education, especially regarding health-related knowledge, were associated with significant improvements in child health [36]. Generally, policymakers emphasize the importance of primary education as compulsory. However, this study emphasizes that females should receive at least secondary education, which could positively influence their children’s nutritional outcomes. Higher levels of education provide women with health awareness and opportunities to participate as laborers in economic activities, contributing to the overall socioeconomic well-being of their households. Maternal involvement in economic activities (employment status) and their education can influence the nutritional outcomes of their children. Educated women are more independent and empowered in society and households, which can positively impact their children’s development. Previous studies also corroborate these findings [37,38,39].

Furthermore, this study has emphasized the importance of sanitation facilities and access to improved water sources in reducing the risk of stunting, being underweight, and wasting. Empirical evidence from various regions in Africa and Asia supports this assertion, indicating that access to clean and safe water helps to decrease the incidence of diseases, mortality rates, and child undernutrition [40]. For example, in households where access to improved water and sanitation was limited among children in Central Africa, specifically Cameroon, the probability of diarrhea was higher [41]. In West Africa, specifically Ghana, the prevalence of stunting in children was reduced by 15% in households with good hygiene and improved water and sanitation [42]. Additionally, the risk of stunting in children was three times greater among households with poor toilets and drinking water quality in Indonesia [43]. In households in India with inadequate sanitation and water, children achieved lower dietary standards by 2% [44]. Similarly, low birth weight has been associated with poor water and sanitation facilities in India [45]. Importantly, the results indicated that through stunting, poor water, sanitation, and hygiene (WASH) negatively affected the children’s development [46]. Some evidence in Pakistan supports the findings of this study, indicating that better water and sanitation facilities contribute to improved nutritional outcomes for children [34,35,47,48].

The interaction analysis suggests that the employment status of women in the first three wealth quintiles did not significantly contribute to the nutritional status of their offspring. In the last two wealth quintiles (richer and richest), maternal employment status did not have a significant effect on the children’s nutritional well-being compared to household wealth status, as both the employed and unemployed mothers contributed to improved child nutrition in the richer and richest quintiles. This outcome aligns with findings from a previous study [49].

The logistic regression analysis revealed that maternal educational attainment, access to improved sources of drinking water, and enhanced sanitation were associated with a reduced likelihood of child malnutrition. Conversely, variables such as the child’s age, residing in regions such as Sindh, Khyber Pakhtunkhwa (KPK), or Balochistan, and recent episodes of diarrheal illness were linked to increased risks of poor health and malnutrition among children. This investigation has highlighted the crucial role played by maternal education in mitigating child malnutrition. Mothers who attain higher levels of education, particularly at the secondary and postsecondary levels, have a significant and positive impact on the nutrition and well-being of their children. Educated mothers possess enhanced capabilities not only in caring for their children’s well-being but also in engaging in income-generating activities that contribute to the overall financial resources of the household. Therefore, promoting female education has emerged as a critical strategy for improving child nutrition outcomes.

Furthermore, insights into the complex relationship among women’s employment, household wealth, and child nutritional status have been gleaned from the valuable perspectives obtained. The results depicted that the odds of stunting, being underweight, and wasting were lower among the children whose mothers had employment and whose household had an average or middle wealth status. In the middle wealth status household’s interaction category, maternal employment had a significant impact on child nutritional outcomes. The results further depicted that the odds of stunting, wasting, and underweight were lower in the richer and richest households when women were not engaged in any employment. In these two interaction categories, household wealth status had a more significant impact compared to women employment status. Furthermore, the chances of stunting, wasting, and being underweight were shown to decrease if the household belonged to the richer and richest wealth status groups when the mother was employed. This showed that maternal employment gives extra support to family resources in the richer and richest households which, in the end, significantly impacts child nutrition. The socioeconomic status of the household has emerged as a significant determinant of child nutritional outcomes, with women’s employment ideally complementing and sustaining this status.

### Strengths and Limitations of the Study

This study made a distinct contribution by investigating the interaction between socioeconomic status and maternal employment status within the context of child undernutrition among children under the age of three in Pakistan. This age group is particularly important in child development, given their increased vulnerability to illnesses and infections, which necessitates heightened care and attention. The findings, derived from a nationally representative dataset, are robust and carry policy implications that can be broadly applied to inform policy solutions. Ultimately, this research has enhanced our understanding of the interplays among maternal employment, household wealth status, and their impact on child nutritional outcomes, thereby contributing to the ongoing discourse in this field. However, a limitation of this study is the cross-sectional use of data, which limited the available variables for comprehensive analysis.

## 5. Conclusions

The study explored the impact of socio-economic factors on stunting, wasting, and underweight conditions among children. The focus was especially on the relationship between maternal employment, household wealth, and undernutrition among children under three in Pakistan. The prevalence of stunting among children under three years of age in Pakistan was 39.40%, underweight prevalence was 27.37%, and 11.88% wasting. In summary, this research has underscored the significant impact of maternal education, access to clean and safe drinking water, and improved sanitation facilities on reducing the chances of stunting, being underweight, and wasting, while age, incidence of diarrhea, and regional contextual factors increase the probability of stunting, underweight, and wasting among children under three in Pakistan. While maternal employment remains a relevant factor, it is evident that the household’s socioeconomic status is essential in shaping the health status of younger children. The study suggests that for optimal effectiveness in improving child nutrition outcomes, women’s employment should actively contribute to and uphold the socioeconomic well-being of the household. Additionally, better household wealth status plays a more significant role in improving the nutritional status of children under three in Pakistan.

## Figures and Tables

**Table 1:** Undernutrition prevalence (%) among children under three by socio-economic characteristics in Pakistan.

Variables	Stunting		Underweight		Wasting	
	%	*p*-Value	%	*p*-Value	%	*p*-Value
Sex of the Child						
Female	48.28	0.18	48.12	0.40	45.11	0.305
Male	51.72		51.88		54.89	
Child’s Age						
Less than 6 months	11.67	*p* < 0.001	16.25	*p* < 0.05	22.56	*p* < 0.001
6–12 months	8.47		14.06		25.56	
13–18 months	12.59		10.31		13.53	
19–24 months	18.76		14.69		9.77	
25–36 months	48.51		44.69		28.57	
Birth’s Order Number						
1	39.82	0.208	34.06	0.57	33.08	0.113
2	27.92		30.31		36.09	
3	23.80		26.25		20.30	
4 or above	8.47		9.38		10.53	
Mother body-mass-index (BMI)						
<18.5 kg/m^2^	86.14	*p* < 0.002	83.23	*p* < 0.001	89.39	0.938
≥18.5 kg/m^2^	39.29		16.77		10.61	
Education level						
Illiterate	75.06	*p* < 0.001	79.38	*p* < 0.001	73.68	*p* < 0.01
Primary	10.07		10.00		9.02	
Secondary/Middle	10.76		7.19		8.27	
Higher	4.12		3.44		9.02	
Residence						
Rural	64.30	*p* < 0.004	66.56	*p* < 0.001	63.16	0.379
Urban	35.70		33.44		36.84	
Regions						
Punjab	10.53	*p* < 0.001	6.56	*p* < 0.001	9.02	*p* < 0.001
Sindh	24.94		30.31		22.56	
KPK	13.96		14.37		20.30	
Balochistan	22.43		29.69		33.08	
Gilgit Baltistan	5.72		1.88		1.50	
Islamabad-Capital	3.20		0.94		0.75	
Azad Jamu and Kahmir	5.95		3.44		3.01	
FATA	13.27		12.81		9.77	
Water Source						
Improved	28.15	*p* < 0.05	29.38	*p* < 0.001	27.07	*p* < 0.05
Un- improved	71.85		70.62		72.93	
Sanitation Facility						
Improved	39.36	*p* < 0.001	39.06	*p* < 0.001	31.58	*p* < 0.05
Un- improved	60.64		60.94		68.42	
Diarrhea						
Yes	73.23	*p* < 0.05	75.0	*p* < 0.001	75.94	*p* < 0.05
No	26.77		25.0		24.06	
Wealth Index						
Poorest	41.19	*p* < 0.001	45.31	*p* < 0.001	33.08	*p* < 0.001
Poorer	28.38		28.12		34.59	
Middle	14.19		15.0		12.78	
Richer	9.38		8.44		12.78	
Richest	6.86		3.12		6.77	
Mother Employment						
Not-Employed	85.13	*p* < 0.01	83.44	*p* < 0.01	87.22	*p* < 0.05
Employed	14.87		16.56		12.78	
Total	39.40		27.37		11.88	

Source: Authors’ estimations.

**Table 2:** Binary logistic regression results for undernutrition prevalence (Stunting, Underweight, and Wasting) among children under three in Pakistan.

Variables	Stunting		Underweight		Wasting	
	Odd Ratios	95% CI	Odd Ratios	95% CI	Odd Ratios	95% CI
Sex of the child	Female (R)					
Male	1.15	(0.89–1.49)	1.08	(0.81–1.44)	0.72	(0.49–1.07)
Child’s age	<6 months (R)					
6–12 months	1.34	(0.84–2.11)	1.22	(0.73–2.02)	1.70	(0.96–3.04)
13–18 months	1.14	(0.72–1.79)	0.80	(0.46–0.46)	0.70	(0.36–1.36)
19–24 months	2.56 ***	(1.61–4.07)	1.64 *	(1.98–2.74)	1.50 *	(1.24–1.47)
25–36 months	1.94 ***	(1.80–3.80)	1.83 **	(1.22–2.76)	1.03 *	(1.33–1.96)
Age^2^ of the child	0.96 *	(0.17–0.63)	0.49 **	(0.31–0.76)	0.23 *	0.29–0.36)
Birth’s Order Number	Birth order number = 1 (R)					
2	1.03	(0.76–1.42)	1.28	(0.89–1.83)	1.73	(1.08–2.78)
3	0.96	(0.67–1.37)	1.31	(0.89–1.92)	1.15	(0.66–2.00)
4 or above	0.75	(0.46–1.23)	1.14	(0.67–1.94)	1.60	(0.79–3.25)
Mother body-mass-index (BMI)	<18.5 kg/m^2^ (R)					
≥18.5 kg/m^2^	0.71	(0.46–1.09)	0.18	(0.27–1.22)	1.07	(0.56–2.03)
Education level	Illiterate (R)					
Primary	0.73	(0.47–1.14)	0.97	(0.60–1.59)	1.00	(0.43–1.73)
Secondary/Middle	0.57 **	(0.42–0.86)	0.56 **	(0.32–0.98)	0.65 *	(0.30–0.38)
Higher	0.32 ***	(0.19–0.55)	0.46 **	(0.21–1.01)	0.86 **	(0.42–0.88)
Residence	Rural (R)					
Urban	1.22	(0.89–1.66)	0.96	(0.67–1.38)	0.91	(0.57–1.46)
Regions	Punjab (R)					
Sindh	2.75 ***	(1.71–4.41)	3.55 ***	(2.02–6.27)	1.75	(1.82–3.73)
KPK	1.73 **	(1.07–2.81)	2.04 **	(1.11–3.74)	1.89 *	(1.88–4.05)
Balochistan	2.98 ***	(1.82–4.91)	3.48 ***	(1.94–6.22)	3.04 **	(1.43–6.45)
Gilgit Baltistan	0.89	(0.46–1.72)	0.43	(0.15–1.17)	0.29	(0.06–1.43)
Islamabad-Capital	0.95	(0.44–2.04)	0.54	(0.14–2.01)	0.26	(0.03–2.12)
Azad Jamu and Kahmir	1.21	(0.65–2.24)	0.74	(0.31–1.74)	0.49	(0.14–1.66)
FATA	1.07	(0.62–1.83)	1.43	(0.75–2.72)	0.75	(0.30–1.86)
Water source	Un-Improved (R)					
Improved	0.65 **	(0.47–0.92)	0.53 ***	(0.37–0.76)	0.70 **	(0.08–0.82)
Sanitation facility	Un-Improved (R)					
Improved	0.55 **	(0.72–0.99)	0.23 **	(0.76–0.86)	0.29 *	(0.35–0.43)
Diarrhea	No (R)					
Yes	1.41 **	(1.04–1.90)	1.15 ***	(1.28–1.62)	1.94 *	(1.49–1.59)
Wealth Index * Mother Employment			Poorest # Not-Employed (R)			
Poorest # Employed	1.67	(0.89–3.09)	1.55	(0.53–4.62)	0.45	(0.17–1.16)
Poorer # Not-Employed	0.82	(0.56–1.17)	0.78	(0.51–1.18)	1.28	(0.74–2.23)
Poorer # Employed	0.94	(0.41–2.12)	0.73	(0.52–1.84)	1.15	(0.38–3.49)
Middle # Not-Employed	0.59	(1.38–0.93)	0.63	(0.29–1.08)	0.84	(0.64–1.73)
Middle # Employed	0.59 **	(0.22–0.64)	0.91 *	(0.37- 0.48)	0.73 ***	(0.40- 0.75)
Richer # Not-Employed	0.41 ***	(0.25–0.69)	0.49 **	(0.25–0.94)	0.86	(0.37–0.45)
Richer # Employed	0.46 *	(0.40–0.32)	0.83 *	(0.20–0.36)	0.61 **	(0.33–0.57)
Richest # Not-Employed	0.35 **	(0.19–0.62)	0.25 **	(0.10–0.58)	0.57 *	(0.20–0.58)
Richest # Employed	0.17 ***	(0.03–0.87)	0.21 **	(0.05–0.21)	0.67 **	(0.06–0.86)
Number of observations = 1093						

References: odds ratios; and confidence intervals. Significance level: *** if *p* < 0.001; ** if *p* < 0.01; * if *p* < 0.05.

**Table 3:** Sensitivity and Specificity for classification of performance of binary logistic regression.

Classification	Indication	Percentage		
		Stunting	Underweight	Wasting
Sensitivity	Pr(+|D)	51.50%	28.48%	3.03%
Specificity	Pr(−|~D)	81.14%	92.87%	99.69%
Positive predictive value	Pr(D|+)	63.90%	60.40%	57.14%
Negative predictive value	Pr(~D|−)	72.07%	77.26%	88.22%
False + rate for true ~D	Pr(+|~D)	18.86%	7.13%	0.31%
False − rate for true D	Pr(−|D)	48.50%	71.52%	96.97%
False + rate for classified +	Pr(~D|+)	36.10%	39.60%	42.86%
False − rate for classified −	Pr(D|−)	27.93%	22.74%	11.78%
Correctly classified		69.48%	75.07%	88.03%

Classified + if predicted Pr(D) ≥ 0.5, True D defined as stunting, underweight, and wasting! = 0, Source: Author estimations.

## Data Availability

This study utilized the secondary data of the Pakistan Demographic and Health Survey 2017–18. Available online at: https://dhsprogram.com/data/dataset/Pakistan_Standard-DHS_2017.cfm?flag=1 (accessed on 14 August 2023).

## References

[1] Cederholm T., Jensen G.L., Correia M.I., Gonzalez M.C., Fukushima R., Higashiguchi T., Baptista G., Barazzoni R., Blaauw R., Coats A.J. (2019). GLIM criteria for the diagnosis of malnutrition–A consensus report from the global clinical nutrition community. J. Cachexia Sarcopenia Muscle.

[2] Ahmed T., Hossain M., Sanin K.I. (2013). Global burden of maternal and child undernutrition and micronutrient deficiencies. Ann. Nutr. Metab..

[3] Müller O., Krawinkel M. (2005). Malnutrition and health in developing countries. Can. Med. Assoc. J..

[4] Zhang Y.-Q., Li H., Wu H.-H., Zong X.-N. (2021). Stunting, wasting, overweight and their coexistence among children under 7 years in the context of the social rapidly developing: Findings from a population-based survey in nine cities of China in 2016. PLoS ONE.

[5] Wali N., Agho K., Renzaho A.M.N. (2019). Past drivers of and priorities for child undernutrition in South Asia: A mixed methods systematic review protocol. Syst. Rev..

[6] Akhtar S. (2016). Malnutrition in South Asia—A critical reappraisal. Crit. Rev. Food.

[7] World Health Organization (2019). Nutrition Landscape Information System (NLIS) Country Profile Indicators: Interpretation Guide.

[8] McDonald C.M., Olofin I., Flaxman S., Fawzi W.W., Spiegelman D., Caulfield L.E., Black R.E., Ezzati M., Danaei G. (2013). Nutrition Impact Model Study. The effect of multiple anthropometric deficits on child mortality: Meta-analysis of individual data in 10 prospective studies from developing countries. Am. J. Clin. Nutr..

[9] Poirier M.J.P., Grépin K.A., Grignon M. (2020). Approaches and alternatives to the wealth index to measure socioeconomic status using survey data: A critical interpretive synthesis. Soc. Indic. Res..

[10] Brooks-Gunn J., Klebanov P., Liaw F.-r., Duncan G. (2021). Toward an understanding of the effects of poverty upon children. Children of Poverty.

[11] Siponen S.M., Ahonen R.S., Savolainen P.H., Hämeen-Anttila K.P. (2011). Children’s health and parental socioeconomic factors: A population-based survey in Finland. BMC Public Health.

[12] Fernald L.C., Neufeld L.M. (2007). Overweight with concurrent stunting in very young children from rural Mexico: Prevalence and associated factors. Eur. J. Clin. Nutr..

[13] Keino S., Plasqui G., Ettyang G., van den Borne B. (2014). Determinants of stunting and overweight among young children and adolescents in sub-Saharan Africa. Food Nutr. Bull..

[14] Zhang N., Bécares L., Chandola T. (2016). Patterns and determinants of double-burden of malnutrition among rural children: Evidence from China. PLoS ONE.

[15] Asim M., Nawaz Y. (2018). Child malnutrition in Pakistan: Evidence from literature. Children.

[16] Shahid M., Ahmed F., Ameer W., Guo J., Raza S., Fatima S., Qureshi G.M. (2022). Prevalence of child malnutrition and household socioeconomic deprivation: A case study of marginalized district in Punjab, Pakistan. PLoS ONE.

[17] Shahid M., Ameer W., Malik N.I., Alam M.B., Ahmed F., Qureshi M.G., Zhao H., Yang J., Zia S. (2022). Distance to Healthcare Facility and Lady Health Workers’ Visits Reduce Malnutrition in under Five Children: A Case Study of a Disadvantaged Rural District in Pakistan. Int. J. Environ. Res. Public Health.

[18] Cunningham K., Headey D., Singh A., Karmacharya C., Rana P.P. (2017). Maternal and Child Nutrition in Nepal: Examining drivers of progress from the mid-1990s to 2010s. Glob. Food Secur..

[19] Hoddinott J., Maluccio J., Behrman J.R., Martorell R., Melgar P., Quisumbing A.R., Yount K.M. (2011). The Consequences of Early Childhood Growth Failure over the Life Course.

[20] Shekar M., Dayton Eberwein J., Kakietek J. (2016). The costs of stunting in South Asia and the benefits of public investments in nutrition. Matern. Child Nutr..

[21] Horton S., Steckel R.H. (2013). Malnutrition: Global economic losses attributable to malnutrition 1900–2000 and projections to 2050. How Much Have Global Problems Cost the Earth? A Scorecard from 1900 to 2050.

[22] Hoddinott J., Alderman H., Behrman J.R., Haddad L., Horton S. (2013). The economic rationale for investing in stunting reduction. Matern. Child Nutr..

[23] Glick P. (2022). Women’s Employment and Its Relation to Children’s Health and Schooling in Developing Countries: Conceptual Links, Empirical Evidence, and Policies. Cornell Food and Nutrition Policy Program Working Paper.

[24] Becker G.S. (1965). A Theory of the Allocation of Time. Econ. J..

[25] Strauss J., Thomas D., Behrman J.B., Srinivasan T.N. (1995). Human Resources: Empirical Modeling of Household and Family Decisions. Handbook of Development Economics.

[26] World Health Organization (WHO) (2009). Child Growth Standards and the Identification of Severe Acute Malnutrition in Infants and Children: A Joint Statement by the World Health Organization and the United Nations Children’s Fund.

[27] Alam M.B., Shahid M., Alzghoul B.I., Yang J., Zakar R., Malik N.I., Bibi A., Tang K. (2023). The Effects of Financial Stress and Household Socio-Economic Deprivation on the Malnutrition Statuses of Children Under Five during the COVID-19 Lockdown in a Marginalized Region of South Punjab, Pakistan. Children.

[28] Das S., Rahman R.M. (2011). Application of ordinal logistic regression analysis in determining risk factors of child malnutrition in Bangladesh. Nutr. J..

[29] Shahid M., Cao Y., Ahmed F., Raza S., Guo J., Malik N.I., Rauf U., Qureshi M.G., Saheed R., Maryam R. (2022). Does mothers’ awareness of health and nutrition matter? A case study of child malnutrition in marginalized rural community of Punjab, Pakistan. Front. Public Health.

[30] Shahid M., Liu Y., Ameer W., Qureshi M.G., Ahmed F., Tang K. (2022). Comparison of Different Nutritional Screening Approaches and the Determinants of Malnutrition in Under-Five Children in a Marginalized District of Punjab Province, Pakistan. Children.

[31] Chatterjee M., Lumbert J. (1989). Women and nutrition: Reflection from India and Pakistan. Food Nutr. Bull..

[32] Khaliq A., Wraith D., Miller Y., Nambiar-Mann S. (2021). Prevalence, Trends, and Socioeconomic Determinants of Coexisting Forms of Malnutrition Amongst Children under Five Years of Age in Pakistan. Nutrients.

[33] Khan S., Zaheer S., Safdar N.F. (2019). Determinants of stunting, underweight and wasting among children < 5 years of age: Evidence from 2012–2013 Pakistan demographic and health survey. BMC Public Health.

[34] Shahid M., Cao Y., Shahzad M., Saheed R., Rauf U., Qureshi M.G., Hasnat A., Bibi A., Ahmed F. (2022). Socio-Economic and Environmental Determinants of Malnutrition in under Three Children: Evidence from PDHS-2018. Children.

[35] Saheed R., Shahid M., Wang J., Qureshi M.G., Sun X., Bibi A., Zia S., Tang K. (2022). Impact of Drinking Water Source and Sanitation Facility on Malnutrition Prevalence in Children under Three: A Gender-Disaggregated Analysis Using PDHS 2017–18. Children.

[36] Fakir A.M.S., Khan M.W.R. (2015). Determinants of malnutrition among urban slum children in Bangladesh. Health Econ. Rev..

[37] Ahmed F., Malik N.I., Shahzad M., Ahmad M., Shahid M., Feng X.L., Guo J. (2022). Determinants of Infant Young Child Feeding Among Mothers of Malnourished Children in South Punjab, Pakistan: A Qualitative Study. Front. Public Health.

[38] Ahmed F., Malik N.I., Malik N., Qureshi M.G., Shahzad M., Shahid M., Zia S., Tang K. (2022). Key Challenges to Optimal Therapeutic Coverage and Maternal Utilization of CMAM Program in Rural Southern Pakistan: A Qualitative Exploratory Study. Nutrients.

[39] Shafiq A., Hussain A., Asif M., Hwang J., Jameel A., Kanwel S. (2019). The effect of “women’s empowerment” on child nutritional status in Pakistan. Int. J. Environ. Res. Public Health.

[40] Anand A., Roy N. (2016). Transitioning toward sustainable development goals: The role of household environment in influencing child health in Sub-Saharan Africa and South Asia using recent demographic health surveys. Front. Public Health.

[41] Dharod J.M., Nounkeu C.D., Paynter L., Labban J.D., Sastre L.R. (2021). Examination of the Cameroon DHS data to investigate how water access and sanitation services are related to diarrhea and nutrition among infants and toddlers in rural households. J. Water Health.

[42] Azupogo F., Abizari A.R., Aurino E., Gelli A., Osendarp S., Bras H., Brouwer I.D. (2020). Malnutrition, hypertension risk, and correlates: An analysis of the 2014 ghana demographic and health survey data for 15–19 years adolescent boys and girls. Nutrients.

[43] Torlesse H., Cronin A.A., Sebayang S.K., Nandy R. (2016). Determinants of stunting in Indonesian children: Evidence from a cross-sectional survey indicate a prominent role for the water, sanitation and hygiene sector in stunting reduction. BMC Public Health.

[44] Choudhary N., Schuster R., Brewis A., Wutich A. (2020). Water insecurity potentially undermines dietary diversity of children aged 6–23 months: Evidence from India. Matern. Child Nutr..

[45] Baker K.K., Story W.T., Walser-Kuntz E., Zimmerman M.B. (2018). Impact of social capital, harassment of women and girls, and water and sanitation access on premature birth and low infant birth weight in India. PLoS ONE.

[46] Ngure F.M., Reid B.M., Humphrey J.H., Mbuya M.N., Pelto G., Stoltzfus R.J. (2014). Water, sanitation, and hygiene (WASH), environmental enteropathy, nutrition, and early child development: Making the links. Ann. N. Y. Acad..

[47] Ahmed F., Shahid M., Cao Y., Qureshi M.G., Zia S., Fatima S., Guo J. (2021). A Qualitative Exploration in Causes of Water Insecurity Experiences, and Gender and Nutritional Consequences in South-Punjab, Pakistan. Int. J. Environ. Res. Public Health.

[48] Jabeen S., Mahmood Q., Nawab B. (2020). High economic impacts of poor water and sanitation in various communities in Pakistan (an environmental economic perspective). Cent. Asian J. Environ. Sci. Technol. Innov..

[49] Shahid M. (2020). Interaction of Household Wealth and Women Working Status on Child Malnutrition: Evidence from PDHS-2013. Pak. Perspect. J..

